# Cine magnetic resonance imaging predicts thrombus adhesion in metastatic renal cell carcinoma with an inferior vena cava tumor thrombus: A case of pathological complete response with pembrolizumab plus lenvatinib

**DOI:** 10.1002/iju5.12668

**Published:** 2023-11-08

**Authors:** Takuto Hara, Junya Furukawa, Takahiro Tsutiya, Takayuki Kodama, Keiichiro Uehara, Tomoaki Terakawa, Kenichi Harada, Jun Teishima, Yuzo Nakano, Masato Fujisawa

**Affiliations:** ^1^ Department of Urology Kobe University Graduate School of Medicine Kobe Japan; ^2^ Department of Diagnostic Pathology Kobe University Graduate School of Medicine Kobe Japan; ^3^ Department of Urology University of Occupational and Environmental Health Kitakyushu Japan

**Keywords:** cine MRI, lenvatinib, pathological complete response, pembrolizumab, renal cell carcinoma

## Abstract

**Introduction:**

Renal cell carcinoma with an inferior vena cava tumor thrombus is a challenging disease that requires a multimodal treatment approach. Pembrolizumab plus lenvatinib has displayed promising efficacy in metastatic renal cell carcinoma.

**Case presentation:**

A 61‐year‐old man was diagnosed with metastatic renal cell carcinoma and a tumor thrombus adhering to the inferior vena cava wall by cine magnetic resonance imaging. After 6 months of pembrolizumab and lenvatinib therapy, tumor shrinkage was detected, excluding the advanced portion of the inferior vena cava thrombus, and nephrectomy and thrombectomy were performed. Adhesion of the tumor thrombus to the inferior vena cava wall was observed during surgery. Resection produced a remarkable pathological complete response with no viable cells in the resected specimens, including the thrombus site.

**Conclusion:**

This case highlights the potential of pembrolizumab plus lenvatinib for treating advanced renal cell carcinoma with an inferior vena cava thrombus and the utility of cine magnetic resonance imaging for evaluating thrombus adhesion to the inferior vena cava.

**Abbreviations & Acronyms**
ccRCC: clear cell renal cell carcinoma
HE: hematoxylin and eosin
IMDC: International Metastatic Renal Cell Carcinoma Database Consortium
IVC: inferior vena cava
MRI: magnetic resonance imaging
pCR: pathological complete response
RCC: renal cell carcinoma
TKI: tyrosine kinase inhibitor

**Keynote message**

Our case presents the remarkable story of a metastatic RCC patient with an IVC thrombus who achieved a pathological complete response to the combination of pembrolizumab and lenvatinib. Notably, cine MRI was able to predict thrombus adhesion to the IVC wall prior to surgical resection. These findings pave the way for more effective and less invasive treatments for this challenging disease.

## Introduction

RCC occasionally forms an IVC tumor thrombus, a challenging complication that requires multimodal treatment.^1^ Pembrolizumab, an ICI targeting programmed death receptor‐1, and lenvatinib, a multitargeted TKI, have displayed promising efficacy in metastatic RCC.^2^, ^3^ In this study, we describe a patient with metastatic RCC and an IVC thrombus who experienced a pCR following treatment with pembrolizumab plus lenvatinib.

## Case presentation

**Fig. 1:**
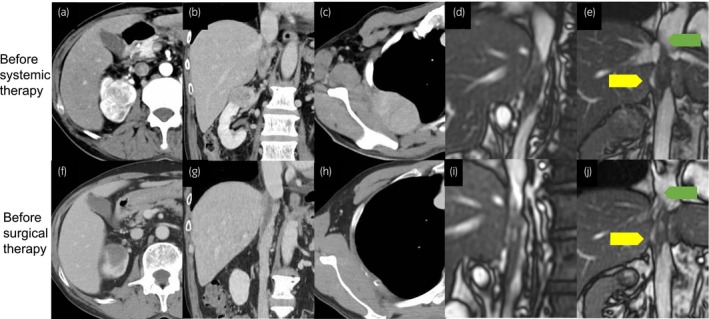
Imaging manifestations of renal cancer, the IVC thrombus, and the fourth rib metastases. (a–c) Computed tomography before systemic therapy. (d, e) MRI before surgery. (f–h) Computed tomography before systemic therapy. (i, j) MRI before surgery. The yellow arrows denote the blood flow between the IVC wall and thrombus, and the green arrows denote the absence of blood flow between the wall and the advanced portion.

A 61‐year‐old man was referred to our hospital after an incidental finding of renal cancer and metastasis to the fourth rib on computed tomography. Further evaluation revealed that the IVC tumor thrombus extended beyond the hepatic venous inflow site to immediately below the diaphragm. This was a Level IIIc thrombus based on Ciancio's classification^4^ (Fig. 1a–c). Cine MRI, an imaging method that specializes in capturing the motions of objects,^5^, ^6^ revealed some blood flow between the IVC wall and the body of the thrombus but no mobility or blood flow between the IVC wall and the advanced portion of the tumor, indicating the possibility of adhesion to or invasion of the IVC wall (Fig. 1d,e).

**Fig. 2:**
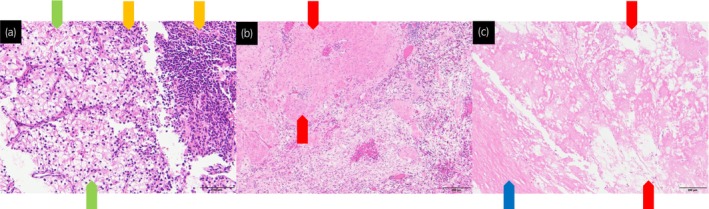
Pathological examination of ccRCC. (a) HE staining (×4) of the renal biopsy specimen prior to systemic therapy. (b) HE staining (×4) of the tumorous area of the resected specimen. (c) HE staining (×10) of the advanced portion of the thrombus in the surgically resected specimens. The yellow arrows denote inflammatory cell infiltration, the green arrows denote ccRCC, the red arrows denote the necrotic area of the tumor, and the blue arrow denotes the IVC wall.

A pretreatment tumor biopsy revealed clear cell RCC with inflammatory cell infiltration on HE staining (Fig. 2a).

The patient was treated with pembrolizumab every 3 weeks and lenvatinib every day for 6 months, resulting in tumor shrinkage (Fig. 1f–h). The rib metastases shrank from 56 to 36 mm, and the main body of the renal tumor was reduced from 45 to 25 mm in the transverse section. The advanced portion of the IVC thrombus remained under the diaphragm, and its size was generally unchanged. The findings on preoperative cine MRI were similar to those before systemic therapy, including some blood flow between the IVC wall and the thrombus but no mobility or blood flow between the wall and advanced portion (Fig. 1g,i).

During the course of treatment, hypertension, diarrhea, and stomatitis were observed, requiring a dose reduction of lenvatinib from 20 to 14 mg on Day 11 of treatment. His number of risk factors improved from two before systemic therapy to one after 6 months from the initiation of therapy, based on the International Metastatic RCC Database Consortium risk classification^7^ (Table 1). Given the improvement in metastatic site and IMDC risk, a decision was made to proceed with nephrectomy at this juncture.

**Table 1:** Patient clinical informations

	Before systemic therapy	Before surgical therapy	
Karnofsky performance status	100	100	%
Diagnosis to systemic therapy	<1 year†	<1 year†	
White blood cell	5900	4400	/μL
Neutrophil	77.2	66.9	%
Lymphocyte	17.6	24.9	%
Neutrophil–lymphocyte ratio	4.39	2.69	
Hemoglobin	12.1†	13.8	g/dL
Platelet	30.6	34.3	×10^4^/μL
Serum calcium	9.7	9.7	mg/dL
Serum albumin	4.2	4.8	g/dL
C‐reactive protein	0.39	0.08	mg/dL
Lactate dehydratase	170	172	U/L
Number of factors meeting IMDC risk classifications	2	1	Factors

† The factor meeting IMDC risk classifications.

The patient underwent open nephrectomy plus thrombectomy after the withdrawal of lenvatinib 7 days prior to surgery. Surgery was initiated through a midline abdominal incision. Perirenal adhesions were mild, with scant evidence of preoperative systemic therapy. The liver was completely mobilized to expose the hepatic vein via incision of the falciform, triangular, and coronary ligaments. Vascular cramps were made superior to the hepatic veins, the left renal vein, and the IVC immediately below it. Then the “Pringle maneuver” (temporarily clamping the portal vein and hepatic artery)^8^ was performed. Cavotomy and thrombectomy were then performed under direct vision. The tumor thrombus was found adherent to the IVC wall in multiple locations. A 6‐cm cavotomy and thrombus removal were performed. In order to shorten the time of the Pringle maneuver, the cardiovascular surgeon simply performed a continuous suture of the IVC defect as planned preoperatively. The surgical time was 434 min, and the blood loss was 135 mL.

The pathological examination of the resected renal tumor and IVC thrombus revealed no viable tumor cells, indicative of a remarkable pCR (Fig. 2b,c).

He has undergone follow‐up without further treatment on the fourth rib metastases for 5 months.

## Discussion

In this study, the use of pembrolizumab plus lenvatinib led to a pCR in a patient with an IVC thrombus. To our knowledge, this is the first such reported case.

The invasiveness of surgery to excise an IVC thrombus varies greatly depending on the height of the advanced site. IVC thrombus categorized as level III or higher according to Ciancio's classification^4^ might requires liver decompensation or the “Pringle maneuver,” as in the present case. Level IV thrombus require open chest surgery and extracorporeal circulation. In addition to increased surgical invasiveness, increased complication and perioperative mortality rates have been reported for this strategy,^9^ and the possibility of reducing surgical invasiveness via preoperative systemic therapy has been assessed.

Drugs suitable for preoperative systemic therapy have a low progressive disease rate, a good overall response rate, a rapid therapeutic effect, and few adverse events,^10^ but to date, drugs with these characteristics have not been identified for treating RCC with an IVC thrombus.

Reports of pCR in the primary lesion in patients who underwent nephrectomy after first‐line immunotherapy‐based combination regimens are increasing. As shown in Table 2,^11^, ^12^, ^13^, ^14^, ^15^, ^16^ pCR with these novel therapies has been reported in 5.3–30% of patients. However, most of the reports are for ipilimumab plus nivolumab therapy, and the pCR rates for immunotherapy plus TKI are unclear. Furthermore, no previous reports have delineated pCR rates exclusive to IVC thrombus cases. Although no studies have investigated whether systemic therapy should be continued in patients who have achieved pCR in primary lesions, systemic therapy was discontinued in this case following consultation with the patient.

**Table 2:** pCR rate of primary kidney lesions

Reports (year)	Therapeutic agents	Number of patients	pCR cases (%)
Reimers *et al*. (2020)^11^	Ipilimumab + nivolumab	5	1 (20.0)
Shirotake *et al*. (2022)^12^	Ipilimumab + nivolumab	10	3 (30.0)
Yoshino *et al*. (2022)^13^	Ipilimumab + nivolumab	7	2 (28.6)
Meerveld‐Eggink *et al*. (2022)^14^	Ipilimumab + nivolumab	13	1 (7.7)
Panian *et al*. (2023)^15^	Nivolumab Nivolumab + cabozantinib Ipilimumab + nivolumab	52	7 (13.5)
Yip *et al*. (2023)^16^	Ipilimumab + nivolumab Pembrolizumab + axitinib	113	6 (5.3)

Cine MRI has been reported to be effective in evaluating adhesions in the abdomen and lungs, and we previously reported its effectiveness in patients with renal cancer and IVC thrombus.^5^, ^6^, ^17^ In that previous report, we indicated that the absence of mobility or blood flow between the IVC wall and the thrombus predicts adhesion with a sensitivity of 100% and a specificity of 85.7%.^17^ In the present case, shrinkage of the primary tumor and metastases was observed, resulting in a pCR, but no shrinkage of the advanced portion of the thrombus was achieved. We believe that this might have been influenced by the adhesion of the thrombus to the IVC wall.

## Conclusion

We report a case of metastatic renal cancer with a tumor thrombus of the IVC that was treated with pembrolizumab plus lenvatinib and deferred cytoreductive nephrectomy, which resulted in a pCR. Cine MRI was useful in predicting thrombus adhesions to the IVC.

## Author contributions

Takuto Hara: Conceptualization; data curation; formal analysis; investigation; methodology; writing – original draft; writing – review and editing. Junya Furukawa: Writing – original draft; writing – review and editing. Takahiro Tsutiya: Data curation; formal analysis. Takayuki Kodama: Formal analysis; investigation. Keiichiro Uehara: Formal analysis; investigation. Tomoaki Terakawa: Writing – original draft; writing – review and editing. Kenichi Harada: Writing – original draft; writing – review and editing. Jun Teishima: Writing – original draft; writing – review and editing. Yuzo Nakano: Writing – original draft; writing – review and editing. Masato Fujisawa: Supervision.

## Conflict of interest

The authors declare no conflict of interest.

## Approval of the research protocol by an Institutional Reviewer Board

No ethics approval was required for this case report.

## Informed consent

Written informed consent was obtained from the patient.

## Registry and the Registration No. of the study/trial

Not applicable.
